# Successful Surgical Management of Locally Advanced Renal Cell Carcinoma Invading Spleen and Pancreas

**DOI:** 10.15586/jkcvhl.v9i3.231

**Published:** 2022-08-12

**Authors:** Mohamed Sharafeldeen, Vahid Mehrnoush, Asmaa Ismail, Ahmed Zakaria, Hazem Elmansy, Walid Shahrour, Owen Prowse, Ahmed Kotb

**Affiliations:** 1 Urology Department, Alexandria University, Alexandria, Egypt;; 2 Urology Department, Northern Ontario School of Medicine, Thunder Bay, ON, Canada

**Keywords:** RCC, nephrectomy, splenectomy, pancreatectomy

## Abstract

Over the last two decades, the treatment of metastatic RCC has changed significantly, and the role of surgery is being debated. A 50-year-old man presented with pain in his left loin. An ultrasound, followed by a CT scan, revealed a 17.5 cm left renal mass invading the left suprarenal gland, spleen, and pancreatic tail. Radical nephrectomy through chevron incision under epidural block with general anesthesia was performed. The entire mass was removed en bloc. The estimated blood loss was 300 mL, and no blood transfusions were performed. The operation took approximately 2 h. Histological examination revealed clear cell renal carcinoma with extension into the spleen, pancreatic tail, and diaphragmatic fibers with negative resection margin. The patient discharged after a 3-day uneventful hospital stay. Aggressive surgical removal of a locally invasive renal cell carcinoma is feasible and should be considered in patients with good performance status and no or minimal distant metastases.

## Introduction

Renal cell carcinoma (RCC) is the sixth and tenth most common cancer and accounting for 5 and 3% of all oncological diagnoses in men and women, respectively (1). Smoking, obesity, and hypertension are those risk factors strongly associated with RCC (2). Although the majority of detected lesions are small tumors, a significant proportion of patients are diagnosed with locally advanced disease, with up to 17% of patients having distant metastases at the time of diagnosis (3). The most common metastasis sites are the lung(s) (71%), lymph nodes (49%), bone (36%), liver (21%), adrenal (9%), brain (9%), pancreas (5%), pleura (4%), and thyroid (0.6%) (4). Although uncommon, isolated metastatic RCC to the pancreas or spleen has been described in the literature (4–7); however, only a few cases of RCC invading both the spleen and the pancreas have been reported (8, 9).

The treatment options for locally advanced RCC vary; including cytokine-based immunotherapy and angiogenic drugs (10–12) and aggressive surgical management (12, 13). Some urologists strongly opposed surgical management due to the surgical challenge and the risk of recurrence (14). We present a case of locally advanced RCC with spleen and pancreatic tail invasion that was successfully treated with surgical intervention.

### Case Presentation

**Figure 1:**
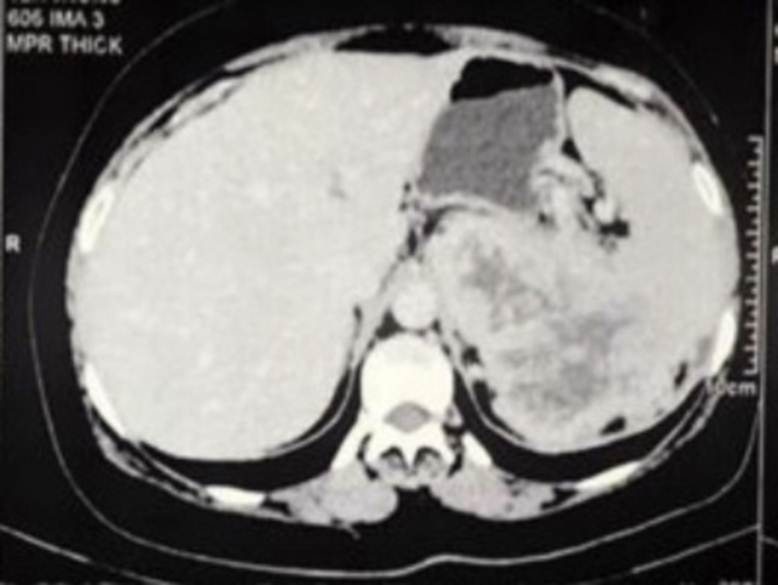
Axial CT abdomen showing large left renal mass infiltrating the spleen and pancreas.

**Figure 2:**
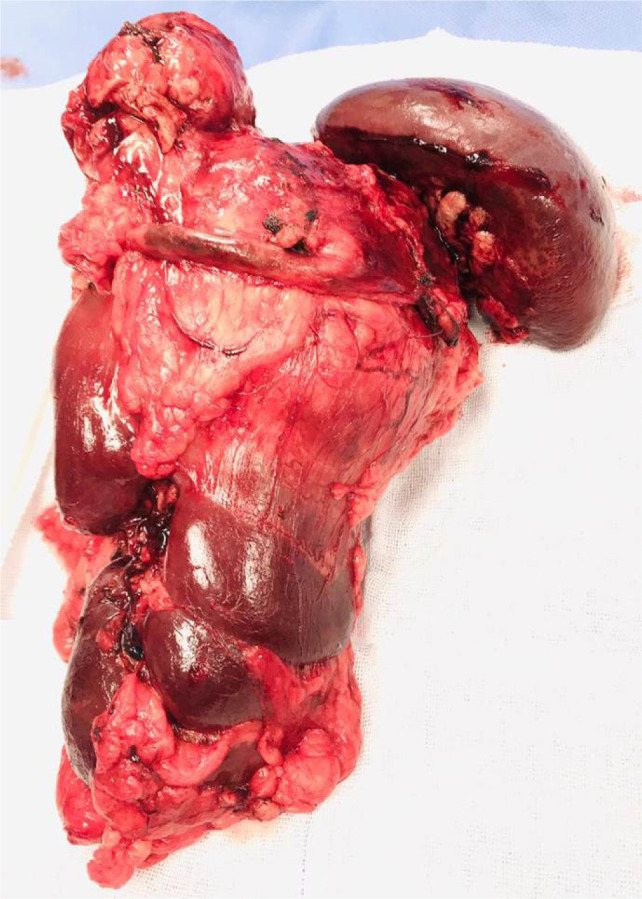
The resected en bloc mass showing the kidney, spleen, and tail of pancreas.

A 50-year-old man presented with left loin pain. An ultrasound, followed by a CT scan, revealed a 17.5 cm left renal mass invading the left suprarenal gland, spleen, pancreatic tail, and a part of the diaphragm. He had excellent performance status and no distant metastases. Radical nephrectomy through chevron incision under epidural block with general anesthesia was performed. Over a 2-hour operation, the entire mass was removed including the kidney, adrenal gland, spleen, pancreatic tail, and diaphragmatic fibers. There was a large chain of para-aortic lymph nodes that was removed as well. The estimated blood loss was 300 mL, and no blood transfusions were required. Chest tube was inserted and removed after 48 h. Histological examination revealed Grade 4 clear cell RCC with extension into the spleen, pancreatic tail, and diaphragmatic fibers. Invasion of main renal vein and pelvicalyceal system was identified. There was neither sarcomatoid differentiation nor lymphovascular invasion. All surgical margins were negative. The resected lymph nodes were reactive with no evidence for cancer (Stage T4N0). Preoperatively, Hgb was 12 g/dL and eGFR was 85 mmol/L. On the first postoperative day, Hgb was 10 g/dL and eGFR dropped to 60 mmol/L, and these values were nearly stable during hospital admission. Four weeks post operation, the patient was reviewed. His wound had healed well, with no complications. Chest X-ray was normal. Hgb did rise to 13 g/dL, and eGFR was 80 mmol/L. The patient was discharged after a 3-day uneventful hospital stay. Six months’ CT scan did not show any evidence for cancer recurrence. We referred the patient to medical oncology team, and we agreed on surveillance, and systemic therapy was not offered. Within that short term follow-up, the patient recovered well and did not show evidence for residual or recurrent cancer. Patient’s consent was obtained for data collection and publication. Ethical approval was not required. Figure 1 is a CT section showing the advanced renal mass and Figure 2 is an operative image for the resected kidney, spleen and pancreatic tail.

## Discussion

Pancreatic and splenic metastasis of RCC generally occur in the seventh decade of life and are usually asymptomatic (6, 7, 15, 16). However, in our case, the patient was younger and aged 50 years. Splenic metastases from RCC have been reported to be more common in patients with RCC originating in the left kidney, like our case, which is more likely via direct tumor cell spread rather than hematogenous dissemination (15, 16). Clear cell carcinoma is the most common type of renal carcinoma invading the spleen (15–17), which matches our scenario.

Over the last two decades, the treatment of metastatic RCC has changed significantly, and the role of surgery is being debated. In certain clinical situations, surgical resection of metastatic RCC to the pancreas and spleen is appropriate, depending on the virulence of the primary tumor, the spread of metastatic disease, the patient’s condition, and the experience of surgeon (8, 9). The goal of resection should be to achieve clear margins while preserving as much healthy tissues as possible. It has been suggested that metastasectomy during nephrectomy improves survival over nephrectomy alone (18).

Two cases of successful laparoscopic and robotic nephrectomy were reported in the literature. The first case of RCC with invasion to spleen and pancreas was reported in 2012 in a 67-year-old woman presented with pain of the left flank and hematuria. A large mass was observed in the upper pole of the left kidney, with direct extension to the spleen and pancreatic tail, but no distant metastasesin the CT scan. Laparoscopic left nephrectomy along with the distal splenopancreatectomy en bloc with estimated blood loss of 250 mL, was performed successfully in 210 min (8). This case had a follow up of 6 years and showed development of pancreatic adenocarcinoma during follow–up, and this was successfully resected. Another case reported in 2016 was of a 57-year-old woman presented with recurrent gross hematuria as well as a large left renal mass with solitary pancreatic and splenic metastasis. Following a thorough evaluation, urologists and transplant or hepatobiliary teams collaborated to perform an en bloc robot-assisted radical nephrectomy, distal pancreatectomy, and splenectomy. The left kidney, the left adrenal gland, the spleen, and the pancreatic tail were all removed. The total operating time was 194 min. The change in hemoglobin during surgery was 2.3 g/dL with no need for blood transfusion.The patient was discharged on Day 3 post operation, with no postoperative complications. The study, however, did not mention the oncological outcomes or duration for surveillance (9). Piskorz et al. reported their experience in a similar case of RCC invading the pancreas and confirmed better survival with surgical excision. Their patient had 2 years follow-up showing no evidence of recurrence (19). Gonzalez et al. reported their experience on doing extensive surgeries of 18 patients having RCC invading liver, spleen and/or the duodenum. They confirmed that besides being safe and with no associated mortality, it, at least, allowed palliation of symptoms associated with extensive cancer invasion to surrounding organs. They had surveillance postoperative scans for up to 2 years showing no evidence for cancer recurrence (20).

Likewise, in our case, surgical management was achievable, within reasonable time and acceptable outcomes. Within a short period of surveillance (6 months), the patient did not show any evidence for cancer recurrence.

## Conclusion

Surgical management for locally extensive RCC should always be considered by experienced urologists to patients with a good performance status and no or minimal distant metastases.
